# Females Exhibit Greater Peak and Cumulative Patellofemoral Joint Stress With Moderate and Heavy Load Carriage Compared With Males

**DOI:** 10.1002/ejsc.70046

**Published:** 2025-09-04

**Authors:** Richard W. Willy, Janet E. Simon, Brittany Hanser, Marin Plemmons, Kelly Christensen, Lexi Klawitter, Alexis Doutt, Brent C. Ruby

**Affiliations:** ^1^ School of Health and Rehabilitation Sciences The Ohio State University Columbus Ohio USA; ^2^ Department of Athletic Training College of Health Sciences and Professions Ohio University Athens Ohio USA; ^3^ School of Physical Therapy & Rehabilitation Science University of Montana Missoula Montana USA; ^4^ Division of Physical Therapy Emory School of Medicine Emory University Atlanta Georgia USA; ^5^ Montana Center for Work Physiology and Exercise Metabolism School of Integrative Physiology and Athletic Training University of Montana Missoula Montana USA

**Keywords:** biomechanics, gender, injury and prevention, musculoskeletal

## Abstract

Load carriage training is universal during military training, regardless of sex or physical characteristics, and may contribute to the 1.3‐2.2× higher incidence of patellofemoral pain (PFP) in female versus male recruits. This study aimed to assess sex differences in patellofemoral joint (PFJ) stress during load carriage, controlling for anthropometrics and quadriceps strength. Twenty males and 20 females walked (1.35 m/s) on an instrumented treadmill with 0‐kg, 20.4‐kg, and 34.0‐kg of load carriage. An inverse‐dynamics musculoskeletal model estimated peak, impulse, and cumulative PFJ stress. To assess quadriceps strength, peak isometric knee extensor torque normalized to body mass was measured via dynamometry. Analyses of covariance (ANCOVA) adjusting for body mass, height, and quadriceps strength assessed the effects of load (0‐kg, 20.4‐kg, and 34.0‐kg) and biological sex (male, female) on PFJ stress and gait parameters. Females were shorter, had lower mass, and lower quadriceps strength (all *p <* 0.001; *d =* 1.50–1.54, indicating large effect sizes). Peak, impulse per step, and cumulative PFJ stress increased with load carriage, with greater increases in females compared to males (sex × load interactions *p =* 0.002–0.005; *η*_*p*_^2^ *=* 0.12–0.13, indicating moderate effect sizes) after controlling for body mass, height, and quadriceps strength. These data indicate that anthropometrics and quadriceps strength do not explain the substantially greater increases in per step and cumulative PFJ stress in females versus males with load carriage. Female recruits may benefit from targeted prevention efforts, such as slower progressions of load carriage training, either in amount carried or distance trained, to reduce their risk of PFP.

## Summary

- Females were shorter, with lower body mass, and lower absolute‐ and body‐mass normalized‐isometric quadriceps strength.
- Disproportionately greater increases in patellofemoral joint stress were observed in females compared with males when walking with moderate (20.4‐kg) and heavy (34.0‐kg) load carriage, while controlling for height, body mass, and isometric quadriceps strength.
- When walking with the load carriage, shorter step length, shorter stance time, and increased trunk flexion were observed in females compared to males. However, these sex differences were no longer present after adjusting for height, body mass, and isometric quadriceps strength in the analysis.

## 1 Introduction

Patellofemoral pain (PFP) is one of the most common pathologies in trainees in the United Kingdom and United States Armed Services, with an incidence of 9.7–571.4 per 1000 person‐years (Smith et al. 2018). PFP is characterized by pain under and around the patella (Smith et al. 2018; Willy, Hoglund, et al. 2019). Female military trainees are particularly at risk, with a 1.3–2.2 times greater incidence of PFP compared with male military trainees (Boling et al. 2010; Glaviano et al. 2021). Although the exact etiology of PFP is unclear, high levels of PFJ stress (i.e., PFJ stress = PFJ contact force/PFJ contact area) are suggested to contribute to its development (Farrokhi et al. 2011). Unaccustomed, high levels of cumulative PFJ stress likely precipitate the onset of PFP (Willy, Hoglund, et al. 2019) and this effect is more apparent in military trainees than civilians (Smith et al. 2018).

The initiation of training with load carriage, for example, rucksacks and weapons systems, likely results in rapid increases in PFJ stress. Training with heavy load carriage is a standard occupational requirement for Soldiers, but is known to increase the risk of musculoskeletal injury (J. J. Knapik et al. 2013; J. Knapik and Reynolds 2010). In most western countries, including the United Kingdom and United States Armed Services, female personnel are eligible for all military roles, including frontline combat (Gill et al. 2023). Consequently, march speeds and load carriage are based on Soldier role, rather than by biological sex or anthropometrics (i.e., march speeds and load carriage are standardized) and march speeds are also standardized (Wendland et al. 2022).

Anthropometric and strength differences are present between males and females and may contribute to the greater risk of musculoskeletal injuries in female Soldiers. Females generally weigh less compared with males (Massachusetts US Army Natick Soldier RD and E Center Natick 2012), indicating that carrying the same load represents a greater proportion of their body mass (Gill et al. 2023; Reilly et al. 2019). Females often have shorter height and leg lengths, necessitating a greater number of steps (i.e., loading cycles) to cover the same distance as their male counterparts (Gill et al. 2023), potentially leading to greater cumulative PFJ stress with load carriage. The lower quadriceps muscle strength in females compared to males (Allison et al. 2015), further suggests that females rely on a greater proportion of their available muscle force production when carrying identical loads as males. Despite the high rate of PFP in female recruits, the effects of operationally relevant load carriage on sex differences in PFJ stress have not been studied (Gill et al. 2023; Wendland et al. 2022).

There are several important limitations of previous sex‐comparison studies examining load carriage biomechanics. Although military load carriage requirements are standardized, most prior studies scaled loads proportional to participants' body mass (e.g., 15%–40% of body mass) and/or used self‐selected walking speeds (Gill et al. 2023). Furthermore, only three sex‐comparison studies (Martin and Nelson 1986; Bode et al. 2021; D. Vickery‐Howe, Dascombe, et al. 2023) used heavy load carriage (≥ 34‐kg) which is consistent with standardized approach loads typical of advanced military training. The remaining studies used light or moderate loads for female participants, which are more typical of fighting loads (Wendland et al. 2022; J. J. Knapik et al. 2004; U.S. Department of the Army, 1990).

Additionally, considerable overlap exists between males and females with respect to anthropometrics and lower limb strength. Allison et al. (Allison et al. 2015) found that the top quartile of female Soldiers had greater quadriceps strength than the bottom quartile of male Soldiers. Female Soldiers readily improve muscular performance with training, effectively reducing sex differences during tasks such as load carriage training and repetitive lifting (Reilly et al. 2019). Together, these data suggest it is possible that sex differences in PFJ stress with load carriage may be attributed to lower body mass, height, and quadriceps strength rather than true sex differences. For instance, Bode et al. (Bode et al. 2021) found few differences in lower limb kinematics and spatiotemporal metrics during load carriage in height‐ and mass‐matched males and females. No load carriage studies to date have controlled for the potential confounders of anthropometrics and quadriceps strength to clearly determine sex differences in PFJ stress with load carriage. Gaining an understanding of factors contributing to sex differences in PFJ stress with load carriage can inform injury prevention efforts in military trainees.

We sought to determine sex differences in PFJ stress during treadmill walking at a fixed speed with moderate (20.4‐kg) and heavy (34.0‐kg) load carriage, typical of loads carried in the US Army. We hypothesized that females would experience greater peak and cumulative PFJ stress with load carriage compared with males. Because increases in PFJ stress with load carriage may be due to anthropometrics and muscle performance rather than solely due to biological sex, our analyses also controlled for the potential covariates of body mass, height, and quadriceps strength.

## 2 Methods

### 2.1 Participants

This cross‐sectional laboratory study is reported using the STROBE checklist (von Elm et al. 2008). The human subjects research protocol and consent form were approved by the Institutional Human Subjects Research Board (UM IRB Protocol #80‐18). Written and verbal consent was obtained from all participants prior to enrollment. Enrolled participants were between the ages of 18–35, injury‐free for at least the past 90‐day, and free of any history of lower extremity surgeries.

Participants were recruited from local trail running clubs and a university, and were required to have experience with load carriage of at least 20‐kg within the past 3 months. Participants were also required to have a habitual Tegner activity level of ≥ 5/10 (Tegner and Lysholm 1985) since this level of physical activity would be expected of recruits entering basic combat training. Male and female participants were matched for age and Tegner level. Using an *α =* 0.05 and *β =* 0.2, and a moderate effect size (*ηp* ^2^ *=* 0.13) for a within‐ and between‐factors repeated measures analysis of variance (ANOVA) for three load carriage conditions, it was determined that at least 14 participants per group were required to adequately power this investigation (G*Power 3.1.9.6).

### 2.2 Procedures

The limb used to kick a ball was the limb of interest. Quadriceps strength was measured isometrically, which was chosen due to the ease in testing participants who were unfamiliar with more dynamic strength testing, such as isokinetic testing. A Kin‐Com 125AP dynamometer (Isokinetic International, Harrison, TN) was used to isometrically assess quadriceps strength. To do so, the participant was seated on the dynamometer with the knee positioned at 90 degrees of knee flexion, and peak isometric knee extensor torque (Nm) was determined and normalized to body mass (Nm/kg) (Mizner et al. 2008). The best of three maximal isometric efforts were retained for analysis.

For motion analysis trials, participants wore standardized footwear (Pegasus 36, Nike Corporation, Beaverton, OR) and a weight‐vest (V‐MAX, weightvest.com, Rexburg, ID, USA; 1.13‐kg mass when empty). Ankle height footwear was used for testing, versus a military boot, to enable accurate marker placement on the malleoli. A 51‐marker, bilateral lower extremity and trunk marker set was applied to define joint centers, segment coordinate systems and dimensions, and to track segments during motion trials (Willy, Willson, et al. 2016). Marker (200‐Hz, Vicon Nexus, Oxford, UK) and instrumented treadmill data (1000‐Hz, Bertec USA, Worthington, OH) were collected during motion trials and processed in real‐time (MotionMonitor, Innovative Sports Inc., Chicago, Ill).

Each participant completed an eight‐minute treadmill accommodation period followed by collection of an unloaded (0‐kg in the weight vest) walking trial. A treadmill walking speed of 1.35 m/sec (4.86 km/hr) was used for all trials to ensure that participants could safely complete each load carriage level. In a counterbalanced order, load carriage trials (moderate: 20.4‐kg; heavy: 34.0‐kg) were then collected at the same standardized speed. The moderate and heavy load carriage conditions were chosen to replicate fighting and approach loads, respectively, in the UK and US Armed services (J. Knapik and Reynolds 2010). To meet each prescribed load carriage condition, 1.13‐kg cast iron weights were loaded in the fore and aft pockets of the weight‐vest to achieve a symmetrical load. A weight vest was chosen instead of a rucksack to enable accurate marker tracking of the pelvis. Marker and instrumented treadmill data were collected for 60‐s per condition. A three‐minute rest period was provided between each condition, with 1 min of treadmill walking provided to accommodate to each load carriage condition.

### 2.3 Data Processing

A 4^th^ order, Butterworth low‐pass filter with 15‐Hz matched frequency cutoffs for kinematic and instrumented treadmill data. Matched filtering cutoff frequencies were used to minimize non‐physiological signal artifacts during inverse dynamics calculations, and > 95% raw signal preservation was confirmed at this cutoff frequency (Kristianslund et al. 2012). Footstrike and toeoff were determined via a 50‐N threshold of the vertical ground reaction force, followed by determination of step length (m) and stance duration (ms). PFJ stress was estimated using a previously described musculoskeletal model (DeVita and Hortobagyi 2001; Willy, Halsey, et al. 2016; Willson et al. 2015) with a custom‐written LabVIEW code (LabVIEW 2023; National Instruments, Austin, TX, USA). Please see Messier et al. (Messier et al. 2011) for a visual representation of the musculoskeletal model. This model estimates gluteus maximus, hamstrings, quadriceps, soleus, and gastrocnemius muscle forces and moments to derive PFJ contact force, which were then used to estimate PFJ stress, and will be briefly described here. First, the proportion of the hip extensor moment attributed to the gluteus maximus and the hamstrings was estimated based on the physiological cross‐sectional area (PCSA) of the respective muscles and moment arms (Ward et al. 2009), varied as a function of hip flexion angle (Nemeth and Ohlsen 1985). Plantarflexor forces were then derived from the quotient of the ankle plantarflexor moment and the Achilles tendon moment arm, varied as a function of ankle angle (Maganaris et al. 2000). Plantarflexor forces were then apportioned to the gastrocnemius and soleus musculature based on the PCSA of each muscle (Ward et al. 2009). Quadriceps muscle forces were then estimated using a two‐step process. First, the knee flexor moment, attributed to the hamstring and gastrocnemius musculature, acting via their respective moment arms at the knee joint, was determined as a function of knee angle (Herzog and Read 1993; Spoor and van Leeuwen 1992; Visser et al. 1990). The knee flexor moment was summed with the internal knee extension moment to yield the quadriceps moment. Next, quadriceps force was determined to be the quotient of the quadriceps moment and the quadriceps moment arm (Herzog and Read 1993; van Eijden et al. 1987). Using quadriceps force, PFJ contact force was calculated as a function of knee angle (van Eijden et al. 1986). PFJ stress was then determined as the quotient of the PFJ contact force and sex‐specific PFJ contact areas, also a function of knee joint angle (Besier et al. 2005). Peak (unit: MPa) and impulse PFJ stress per step (unit: MPA × s) were determined and the maxima and the time integral of the individual PFJ stress curves, respectively. Cumulative PFJ stress (unit: MPa × s/km) was estimated as the product of PFJ impulse per step and the estimated number of steps for the limb of interest to traverse 1‐km (Willy, Willson, et al. 2016), as follows:

(1) limbofintereststepsperkm=(1000m/steplength)/2)

(2) PFJstressperkm=limbofintereststepsperkm∗PFJimpulseperstep

### 2.4 Statistical Analyses

Data were analyzed via SPSS Version 29.0 (IBM, Houston, TX, USA). All data were present with no cases of missing data. Anthropometric and peak isometric knee extensor torque (Nm and Nm/kg) data were analyzed via independent *t*‐tests (*α* = 0.05) and effect sizes were calculated (Cohen's *d*). An analysis of covariance (ANCOVA) was conducted to examine the effects of load (0‐kg, 20.4‐kg, and 34.0‐kg) and sex (male, female) on PFJ stress and related gait parameters, with adjustments for body mass, height, and normalized quadriceps strength to control for individual differences and provide a clearer assessment of the effects of load and sex. ANCOVA assumptions were assessed to validate results. Independence was ensured by design; normality and homogeneity of variances were evaluated via residual plots and Levene's test, respectively. Linearity between covariates and dependent variables was examined with scatterplots, and homogeneity of regression slopes was confirmed. For the within‐subject factor of load (0‐kg, 20.4‐kg, 34.0‐kg), a Greenhouse‐Geisser correction was applied when Mauchly's test indicated sphericity violation. Bonferroni adjustments were applied for pairwise comparisons where appropriate, and statistical significance was set at *p* < 0.05. Partial eta‐squared (*η*_*p*_^2^) effect sizes were calculated for significant interactions and main effects, interpreted as 0.02 for small, 0.13 for medium, and 0.26 for large effects (Bakeman 2005). Temporospatial variables of interest were step length and stance duration. Discrete kinematic variables were peak knee flexion during the first two‐thirds of stance and peak pelvic‐trunk flexion during stance phase. Discrete variables of interest from the musculoskeletal model were peak PFJ stress per step, PFJ stress impulse per step, and PFJ stress impulse per km.

## 3 Results

Females had 16.7% lower body mass, were 5.6% shorter, and 32.3% lower peak isometric knee extensor torque compared with males (all *p <* 0.001, *d =* 0.69–1.54) (Table 1). Females also had 17.8% lower peak isometric knee extensor torque normalized to body mass compared with males (*p =* 0.004; *d =* 0.94).

**TABLE 1:** Demographics, anthropometrics, and isometric peak knee extension torque. Mean (SD).

	Males (*n* = 20)	Females (*n* = 20)	*p*‐value	Cohen's *d*
Age (y)	24.7 (4.1)	24.0 (4.2)	*p* = 0.61	*d* = 0.17
Tegner (x/10)	6.2 (0.9)	5.9 (0.8)	*p* = 0.31	*d* = 0.34
Height (cm)	180.6 (4.8)	170.5 (6.4)	*p* < 0.001	*d* = 1.54
Mass (kg)	77.6 (8.3)	64.7 (8.9)	*p* < 0.001	*d* = 1.50
Quadriceps torque (Nm)	287.4 (65.3)	196.8 (53.5)	*p* < 0.001	*d* = 1.53
Normalized quadriceps torque (Nm/kg)	3.70 (0.71)	3.02 (0.73)	*p* = 0.004	*d* = 0.94

### 3.1 Step Length

Please see Table A1 for the unadjusted means and 95% confidence intervals for gait biomechanics. The ANCOVA revealed no significant interaction between sex and load (*p* = 0.39), no significant main effect of load on step length (*p* = 0.19), nor a significant main effect of sex (*p* = 0.12). Please see Table 2. Given the lack of significant effects, no further pairwise comparisons were conducted.

**TABLE 2:** Results of ANCOVA for temporospatial and kinematic variables of interest. Mean (95% confidence intervals). In the instance of a significant interaction, the main effects are not displayed. Please note these are adjusted means.

		Males	Females	Pooled	*p*‐value
Step length (m)	0‐kg	0.70 (0.69, 0.72)	0.70 (0.68, 0.71)	0.70 (0.69, 0.71)	Sex × load *p* = 0.39 Main effect of sex *p* = 0.12 Main effect of load *p* = 0.19
	20.4‐kg	0.71 (0.69, 0.73)	0.70 (0.68, 0.71)	0.70 (0.69, 0.71)	
	34.0‐kg	0.71 (0.69, 0.73)	0.70 (0.67, 0.71)	0.70 (0.69, 0.71)	
	Pooled	0.71 (0.69, 0.71)	0.69 (0.68, 0.71)	—	
Stance duration (ms)	0‐kg	654.6 (640.2, 669.0)	648.4 (634.1, 662.8)	651.5 (643.1, 660.0)	Sex × load *p* = 0.06 Main effect of sex *p* = 0.22 Main effect of load *p* < 0.001
	20.4‐kg	668.8 (655.0, 682.6)	652.0 (638.2, 665.8)	660.4 (652.3, 668.5)^a^	
	34.0‐kg	674.6 (660.9, 688.4)	657.3 (643.6, 671.0)	665.9 (658.1, 674.0)^a^ ^,^ ^b^	
	Pooled	666.0 (652.6, 679.4)	652.6 (639.1, 666.1)	—	
Peak knee flexion (deg)	0‐kg	17.1 (14.8, 19.5)	15.9 (13.6, 18.3)	16.5 (15.2, 17.9)	Sex × load *p* = 0.64 Main effect of sex *p* = 0.37 Main effect of load *p* = 0.66
	20.4‐kg	18.9 (16.6, 21.3)	17.0 (14.6, 19.3)	18.0 (16.6, 19.4)	
	34.0‐kg	17.9 (15.6, 20.3)	16.3 (13.9, 18.6)	17.1 (15.7, 18.5)	
	Pooled	18.0 (15.8, 20.3)	16.4 (14.1, 18.7)	—	
Peak trunk flexion (deg)	0‐kg	16.3 (12.3, 20.4)	13.9 (9.8, 17.9)	15.1 (12.7, 17.5)	Sex × load *p* = 0.20 Main effect of sex *p* = 0.87 Main effect of load *p* = 0.22
	20.4‐kg	18.0 (12.2, 23.9)	18.7 (12.8, 24.6)	18.4 (14.9, 21.8)	
	34.0‐kg	16.5 (10.7, 22.4)	20.2 (14.3, 26.1)	18.4 (14.9, 21.8)	
	Pooled	17.1 (12.2, 21.7)	17.6 (12.8, 22.4)	—	

*Note:* Covariates appearing in the model are evaluated at the following values: Mass = 71.1‐kg, Quad Strength Normalized = 3.7 Nm/kg, Height = 1.8 m.

^a^ Different from 0 kg (*p* ≤ 0.001).

^b^Different from 20.4 kg (*p* ≤ 0.05).

### 3.2 Stance Duration

The ANCOVA revealed no significant interaction between sex and load (*p* = 0.06), but a significant main effect of load was found for stance duration, F_(2, 75)_ = 10.24, *p* < 0.001, *η*_*p*_^2^ = 0.21, which corresponds to a medium effect size. Stance duration increased across load levels, from 0‐kg (651.5 ms) to 20.4‐kg (660.4 ms, *p* < 0.05) and further at 34.0‐kg (665.9 ms, *p* < 0.001).

### 3.3 Peak Knee Flexion

The ANCOVA revealed no significant interaction effect (*p* = 0.64) and no significant main effects of load (*p* = 0.66) or sex (*p* = 0.37) were observed for peak knee flexion. Thus, no effect size interpretations or further comparisons were necessary. Please also see Table A2 supplemental peak hip flexion and ankle dorsiflexion kinematic data.

### 3.4 Peak Trunk Flexion

The ANCOVA showed no significant sex by load interaction (*p* = 0.20), no significant main effect of load (*p* = 0.22) or sex (*p* = 0.87) on peak trunk flexion, indicating that load and sex had negligible effects on trunk flexion across conditions.

### 3.5 Peak PFJ Stress

**FIGURE 1:**
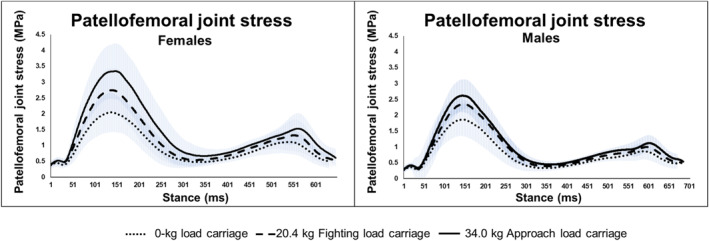
Time series patellofemoral joint stress data (unadjusted values) and standard deviations for female and male participants during each load carriage condition. Data are not time normalized to illustrate sex differences in stance time, with shorter stance times in female participants and increasing stance times with greater load carriage.

The ANCOVA revealed a significant sex × load interaction effect was found for peak PFJ stress between sex and load, F_(2, 75)_ = 5.21, *p* = 0.005, *η*_*p*_^2^ = 0.12, reflecting a medium effect size (Table 3 displays adjusted means; Table A3 displays unadjusted means; Figure 1 displays unadjusted time series data). Pairwise comparisons showed that peak PFJ stress increased significantly at each load level for both sexes, with females consistently exhibiting higher PFJ stress compared to males. For example, at 0‐kg, females had a peak PFJ stress of 2.4 MPa compared to 1.7 MPa for males; at 20.4‐kg, it increased to 3.1 MPa for females and 2.2 MPa for males; and at 34.0‐kg, females reached 3.8 MPa, whereas males were at 2.4 MPa (all comparisons *p* < 0.001).

**TABLE 3:** Results of ANCOVA for patellofemoral joint (PFJ) stress variables of interest. Mean (95% confidence intervals). In the instance of a significant interaction, the main effects are not displayed. Please note these are adjusted means.

		Males	Females	Pooled	*p*‐value
Peak PFJ stress per step (MPa)	0‐kg	1.7 (1.4, 1.9)	2.4 (2.2, 2.7)^d^	2.0 (1.9, 2.2)	Sex × load *p* = 0.005
	20.4‐kg	2.2 (1.9, 2.5)^a^	3.1 (2.8, 3.4)^a^ ^,^ ^d^	2.6 (2.5, 2.8)	
	34.0‐kg	2.4 (2.0, 2.7)^a^	3.8 (3.4, 4.2)^a^ ^,^ ^b^ ^,^ ^d^	3.1 (2.9, 3.3)	
	Pooled	2.1 (1.8, 2.4)	3.1 (2.8, 3.4)	—	
PFJ stress impulse per step (MPa × s)	0‐kg	0.45 (0.38, 0.52)	0.70 (0.62, 0.77)^d^	0.57 (0.54, 0.61)	Sex × load *p* = 0.005
	20.4‐kg	0.57 (0.49, 0.65)^a^	0.85 (0.76, 0.93)^a^ ^,^ ^d^	0.71 (0.66, 0.75)	
	34.0‐kg	0.64 (0.54, 0.75)^a^ ^,^ ^c^	1.0 (0.91, 1.1)^a^ ^,^ ^b^ ^,^ ^d^	0.83 (0.77, 0.88)	
	Pooled	0.55 (0.47, 0.62)	0.86 (0.78, 0.94)	—	
PFJ stress impulse per km (MPa × s/km)	0‐kg	313.2 (270.2, 356.3)	504.4 (461.3, 547.4)^d^	408.8 (383.5, 434.1)	Sex × load *p* = 0.002
	20.4‐kg	394.7 (343.1, 446.4)^a^	615.1 (563.5, 666.7)^a^ ^,^ ^d^	504.9 (474.6, 535.2)	
	34.0‐kg	442.7 (375.4, 509.9)^a^	746.6 (679.3, 813.9)^a^ ^,^ ^b^ ^,^ ^d^	594.7 (555.1, 634.2)	
	Pooled	383.6 (333.4, 433.7)	622.0 (571.9, 672.1)	—	

*Note:* Adjusted Means: Covariates appearing in the model are evaluated at the following values: Mass = 71.1‐kg, Quad Strength Normalized = 3.7 Nm/kg, Height = 1.8 m.

^a^ Different from 0‐kg (*p* < 0.001).

^b^ Different from 20.4‐kg (*p* < 0.001).

^c^ Different from 20.4‐kg (*p* < 0.05).

^d^ Different between sexes at same load carriage level (*p* < 0.001).

### 3.6 PFJ Stress Impulse per Step

The ANCOVA revealed a significant sex by load interaction for PFJ stress impulse per step, F_(2, 75)_ = 5.38, *p* = 0.005, *η*_*p*_^2^ = 0.12, consistent with a medium effect size. Pairwise comparisons indicated a significant increase in PFJ stress impulse per step with increasing load for both sexes. Females exhibited a stress impulse of 0.70 MPa × s at 0‐kg, which rose to 0.85 MPa × s at 20.4‐kg and 1.0 MPa × s at 34.0‐kg, all significantly higher than males at each load level (*p* < 0.001).

### 3.7 PFJ Stress Impulse per Kilometer

The ANCOVA revealed a significant interaction effect for PFJ stress impulse per kilometer, F_(2, 75)_ = 6.59, *p* = 0.002, η_*p*_^2^ = 0.15, indicating a medium effect size. Pairwise comparisons showed that PFJ stress impulse per kilometer increased across load levels for both sexes, with females displaying greater increases than males. Female PFJ stress impulse values rose from 504.4 MPa × s/km at 0‐kg to 615.1 MPa × s/km at 20.4‐kg and 746.6 MPa × s/km at 34.0‐kg, compared to 313.2 MPa × s/km, 394.7 MPa × s/km, and 442.7 MPa × s/km for males (all *p* < 0.001).

## 4 Discussion

This is the first study to our knowledge to assess sex‐differences in PFJ stress during walking with moderate and heavy load carriage. Compared to males, females exhibited greater peaks, impulse per step, and PFJ stress per km. Relative increases in these values were greater in females compared with males with the moderate and heavy load carriage conditions. Notably, sex differences in PFJ stress metrics with load carriage remained after controlling for the body mass, height, and quadriceps strength in females, indicating that other sex‐specific factors may contribute to our findings. Since PFP has been associated with rapid and large increases in peak and cumulative PFJ stress, the introduction of moderate and heavy load carriage to training may particularly increase the risk of PFP in female Soldiers.

The unadjusted analysis (Table A2) revealed disproportionately greater increases in PFJ stress metrics in females compared to males across all load carriage conditions. These differences remained significant after adjusting for body mass, height, and quadriceps strength. One potential explanation is that females have lower PFJ contact area than males which may contribute to the higher PFJ stress observed. Because PFJ stress is defined as the PFJ contact force divided by PFJ contact area, equivalent PFJ contact forces between sexes would still result in higher PFJ stress in females due to the lower PFJ contact area. Additionally, fat‐free mass is lower in female personnel (Roberts et al. 2023) and was not assessed in this study, but may be more closely related to changes in PFJ stress responses with load carriage. For example, fat‐free body mass was found to be the most predictive factor for foot march performance in a recent study in Reserve Officers' Training Corps (ROTC) Cadets (Sax van der Weyden et al. 2024). Although quadriceps weakness is a risk factor for PFP in the military (Neal et al. 2019), our findings suggest it may not relate to PFJ stress with load carriage. Therefore, interventions focusing solely on increasing quadriceps strength may be insufficient to reduce PFJ stress in females undergoing load carriage training.

We still observed greater PFJ stress metrics with load carriage in females after controlling for peak isometric knee extensor torque. There are several possible reasons why controlling for peak isometric knee extensor torque failed to account for sex differences in PFJ stress metrics. First, we assessed peak isometric knee extensor torque, but isokinetic testing of the quadriceps may yield a relationship with PFJ stress that we did not detect. PFJ stress peaks in the first 1/3^rd^ of stance during the loading response as the quadriceps control knee flexion eccentrically. Future testing should evaluate the relationship between isokinetic eccentric quadriceps strength and PFJ stress responses with load carriage. It is also possible that more comprehensive muscle testing may yield stronger relationships with PFJ stress. Specifically, we limited our strength testing to the quadriceps musculature, whereas hip, knee, and ankle musculature are also key contributors to knee biomechanics (Winby et al. 2009). These muscle groups can easily be assessed with dynamometry and future studies should assess the strength of multiple muscle groups to provide insight into mediators of PFJ stress with load carriage.

Most prior load carriage studies used self‐selected walking speeds and/or scaled load carriage to body mass (Gill et al. 2023) which may explain why past studies did not find sex differences in knee extensor moments with load carriage (Gill et al. 2023; Wendland et al. 2022; Middleton et al. 2022; D. M. Vickery‐Howe et al. 2020; Krupenevich et al. 2015). In contrast, we used a standard walking speed and standardized, heavy load carriage. Krupenevich et al. (Krupenevich et al. 2015), as an example, found no sex differences in the peak knee extensor moment with 22‐kg of load at a standardized walking speed. Similar to our study, studies employing standardized walking speeds and standardized, military‐relevant heavy (i.e., ≥ 34‐kg) load carriage also found sex‐differences in walking biomechanics (Martin and Nelson 1986; Loverro et al. 2019). Lastly, Krupenevich et al. assessed the peak knee extensor moment whereas our assessment of PFJ stress accounted for co‐contraction of the knee extensors and flexors, which may also explain the differences between studies.

After controlling for anthropometrics and isometric quadriceps strength, no temporospatial differences were found between males and females during walking with load carriage. However, our unadjusted data (See Table A1) indicated that females took 6% shorter steps (resulting in approximately 39.2 additional steps/km), while also exhibiting a 6% shorter stance duration compared with males. Our adjusted analyses are consistent with Silder et al. (Silder et al. 2013) and Middleton et al. (Middleton et al. 2022) who scaled load carriage to body mass (i.e., 10%–40% of participant body mass) and also did not find sex differences in temporospatial variables. Furthermore, Middleton et al. found that height and mass accounted for nearly 30% of the variance in stance duration, with just 0.1% of the variance explained by sex alone. Bode et al. (Bode et al. 2021) also found no sex differences in temporospatial metrics during load carriage between height‐ and mass‐matched males and females. In contrast to the current study and others, Vickery‐Howe et al., found that stride length was 3.8% greater in females relative to body height, which may be due to their use of faster walking speeds (up to 1.8 m/s) with studies of comparison. The increase in stance duration with load carriage, as found in this investigation, was a near‐universal finding across literature reviewed (Bode et al. 2021; D. Vickery‐Howe, Dascombe, et al. 2023; Middleton et al. 2022), and likely represents a greater proportion of time in double limb support to ensure postural stability with carried loads.

In our unadjusted model (Table A1), we found greater peak trunk flexion in females, similar to Krupenevich et al. who found greater peak trunk flexion in females with 22‐kg of load carriage (Teng and Powers 2014). However, once height, body mass, and quadriceps strength were entered into the model, we no longer found that females exhibited greater peak trunk flexion with load. We also only found a main effect of load (*p* < 0.001) for peak knee flexion in our unadjusted model, but no differences were found once height, body mass, and quadriceps strength were controlled in our adjusted model. Together, these data indicate that any sex‐differences in temporospatial and trunk and knee kinematics can be explained by variances in anthropometrics and quadriceps strength.

Our findings suggest female recruits may benefit from sex‐specific prevention efforts to reduce the risk of PFP when training with load carriage. Since females take shorter steps compared with males (unadjusted data in our investigation), females and (shorter males) may be tempted to increase their step lengths to match taller recruits during load carriage training. However, female and shorter male recruits should be discouraged from this practice since adopting longer step lengths with load carriage will increase peak and cumulative PFJ kinetics further (Willy, DeVita, et al. 2019). Similarly, encouraging a preferred step length with load carriage in female Soldiers reduced their incidence of pelvic bone stress injuries to an equivalent incidence seen in male Soldiers (Pope 1999). Female recruits may also benefit from a slower progression of load carriage training, either in the amount carried or in the distance of load carriage maneuvers, or preparatory training prior to the start of basic combat training to provide more time for sufficient tissue adaption. For instance, completion of a fitness program prior to basic combat training reduced musculoskeletal injury risk in male and female recruits (J. J. Knapik et al. 2006). Additionally, after 6 months of heavy strength training, females achieve load carriage performance comparable to males who have not engaged in strength training (Kraemer et al. 2001). Female Soldiers can also significantly enhance their muscular performance, thereby narrowing the gap in physical performance between sexes during activities such as load carriage and repetitive lifting.

Our study has several strengths and limitations that should be considered. Our subjects fell within 95% of sex‐specific military standard height and body mass [ANSUR‐II] guidelines (Massachusetts US Army Natick Soldier RD and E Center Natick 2012). We enrolled participants who were young, physically active, non‐military personnel with recent load carriage experience. Thus, our sample is representative of those entering basic combat training which is the training phase when military personnel are most at risk for PFP. Secondly, our study controlled for the effects of anthropometrics and quadriceps strength to develop a clearer understanding of sex differences in load carriage biomechanics. We also used standardized walking speed and standardized, military‐relevant, moderate and heavy load carriage to better replicate the load carriage requirements placed on recruits. Our study had several limitations, as well. To facilitate accurate marker placement on the pelvis, we used a weight vest which is a limitation considering that a rucksack is typically used to carry moderate and heavy loads in military training. Other limitations include our use of an instrumented treadmill, which limits applicability to the more varied terrain typical of military training, and walking trials were assessed for 1‐min, per condition. Treadmill walking, while largely similar to overground walking with loads (Fellin et al. 2016), results in a lower peak knee extension moment than overground walking (D. M. Vickery‐Howe, Bonanno, et al. 2023). Prolonged trials with load carriage (Middleton et al. 2022; D. M. Vickery‐Howe et al. 2020), particularly over varied terrain, may yield different results since exertion may affect PFJ kinetics. Downhill walking with load carriage, given its higher quadriceps demands and knee flexion kinematics (Alexander and Schwameder 2016), may reveal relationships between isometric knee extensor torque and PFJ stress metrics that were not found in the current investigation. Lastly, we used a musculoskeletal model that did not use subject‐specific muscle moment arms, cross‐section areas, or PFJ contact areas. All musculoskeletal models have assumptions and known limitations, described fully elsewhere (DeVita and Hortobagyi 2001; Willy, Halsey, et al. 2016; Willson et al. 2015).

## 5 Conclusions

Walking with moderate (20.4‐kg) and heavy (34.0‐kg) load carriage resulted in disproportionately greater increases in per step and cumulative PFJ stress in females compared with males. These data suggest that training with fixed external loads may disproportionately increase the risk of PFP in female recruits compared with males. Considering the elevated risk of PFP in female recruits, these findings may provide important insight for injury prevention programs in military populations.

## Ethics Statement

Prior to initiation of this study, the research protocol was approved by the University of Montana Human Subjects Research Board (UM IRB Protocol #80‐18).

## Consent

Written and verbal consent was obtained from all participants prior to enrollment in this investigation.

## Conflicts of Interest

The authors declare no conflicts of interest.

## Data Availability

The data that support the findings of this study are available from the corresponding author upon reasonable request.

## Appendix A

**TABLE A1:** Supplemental data (Means and 95% confidence intervals). ANOVA for temporospatial and kinematic variables of interest. These are unadjusted data.

	Load carriage	Males (*n* = 20)	Females (*n* = 20)	Pooled mean	*p*‐value
Step length (m)	0‐kg	0.72 (0.70–0.73)	0.68 (0.67–0.70)	0.70 (0.69–0.71)	Sex × load *p* = 0.27 Main effect of sex *p* < 0.002 Main effect of load *p* = 0.80
	20.4‐kg	0.72 (0.70–0.74)	0.68 (0.67–0.70)	0.70 (0.69–0.71)	
	34.0‐kg	0.72 (0.70–0.74)	0.68 (0.66–0.70)	0.70 (0.69–0.71)	
	Pooled mean	0.72 (0.71–0.73)	0.68 (0.67–0.70)^d^		
Stance duration (ms)	0‐kg	677.1 (655.7–686.9)	631.8 (616.1–647.4)	651.5 (640.5–662.6)	Sex × load *p* = 0.94 Main effect of sex *p* < 0.001 Main effect of load *p* < 0.001
	20.4‐kg	680.7 (667.8–693.9)	640.1 (627.1–653.1)	660.4 (651.2–669.6)^b^	
	34.0‐kg	685.6 (672.4–698.7)	646.4 (633.3–659.6)	666.0 (656.7–675.3)^b^ ^,^ ^c^	
	Pooled mean	679.2 (665.7–692.7)	639.4 (625.9–653.0)^d^		
Peak knee flexion (deg)	0‐kg	16.9 (15.0–18.8)	16.2 (14.2–18.1)	16.4 (15.2–17.9)	Sex × load *p* = 0.13 Main effect of sex *p* = 0.40 Main effect of load *p* < 0.001
	20.4‐kg	18.4 (16.3–20.4)	17.6 (15.5–19.7)	18.0 (16.5–19.4)^b^	
	34.0‐kg	18.1 (16.1–20.0)	17.6 (15.5–19.7)	17.1 (15.7–18.5)^a^ ^,^ ^c^	
	Pooled mean	17.8 (15.9–19.7)	16.6 (14.7–18.6)		
Peak trunk flexion during loading response (deg)	0‐kg	15.9 (12.4–19.4)	14.3 (10.8–17.8)	15.1 (12.6–17.5)	Sex × load *p* = 0.006
	20.4‐kg	16.3 (11.5–21.2)	20.4 (15.6–25.3)^a^	18.4 (14.9–21.8)	
	34.0‐kg	15.2 (10.5–20.0)	21.5 (16.7–26.3)^a^	18.4 (15.0–21.8)	
	Pooled mean	15.8 (11.9–19.8)	18.8 (14.8–22.7)		

*Note:* Mean (95% confidence intervals). In the instance of a significant interaction, the main effects are not displayed. Please note unadjusted means and standard deviations are displayed.

^a^ Different from 0‐kg (*p* < 0.05).

^b^ Different from 20.4‐kg (*p* < 0.001).

^c^ Different from 20.4‐kg (*p* < 0.05).

^d^ Different between sexes (*p* < 0.001).

**TABLE A2:** Supplemental data.

	Load carriage	Males (*n* = 20)	Females (*n* = 20)	Pooled mean
Peak ankle dorsiflexion (deg)	0 kg	13.5 (12.2–14.8)	14.8 (13.6–16.1)	14.2 (13.3–15.1)
	20.4 kg	13.6 (12.2–15.0)	14.0 (12.6–15.4)	13.8 (12.8–14.8)
	34.0 kg	13.6 (12.1–15.1)	14.2 (12.7–15.7)	13.9 (12.9–15.0)
	Pooled mean	13.6 (12.2–14.9)	14.4 (13.0–15.7)	
Peak hip flexion (deg)	0 kg	19.9 (16.7–22.9)	21.4 (18.3–24.4)	20.6 (18.4–22.8)
	20.4 kg	20.4 (17.1–23.7)	21.5 (18.2–24.8)	20.9 (18.6–23.3)
	34.0 kg	20.8 (15.6–26.0)	26.1 (21.0–31.4)	23.5 (19.8–27.2)
	Pooled mean	20.3 (16.8–23.9)	23.0 (19.5–26.5)	

*Note:* Mean (95% confidence intervals). Please note these are unadjusted means and standard deviations.

**TABLE A3:** Supplemental data (Means and 95% confidence intervals). ANOVA for patellofemoral joint stress variables of interest. These are unadjusted data.

	Load carriage	Males (*n* = 20)	Females (*n* = 20)	Pooled mean	*p*‐value
Peak PFJ stress per step (MPa)	0‐kg	1.93 (1.68–2.28)	2.13 (1.89–2.38)	2.03 (1.86–2.21)	Sex × load *p* < 0.001
	20.4‐kg	2.41 (2.12–2.71)^a^	2.87 (2.58–3.16)^a^ ^,^ ^d^	2.64 (2.44–2.85)	
	34.0‐kg	2.69 (2.34–3.04)^a^ ^,^ ^c^	3.48 (3.13–3.83)^a^ ^,^ ^b^ ^,^ ^d^	3.09 (2.84–3.33)	
	Pooled mean	2.34 (2.07–2.62)	2.83 (2.55–3.11)		
PFJ stress impulse per step (MPa × s)	0‐kg	0.534 (0.464–0.604)	0.612 (0.543–0.682)	0.573 (0.524–0.623)	Sex × load *p* < 0.001
	20.4‐kg	0.650 (0.570–0.730)^a^	0.767 (0.687–0.847)^a^ ^,^ ^d^	0.708 (0.652–0.756)	
	34.0‐kg	0.728 (0.634–0.822)^a^ ^,^ ^c^	0.929 (0.835–1.023)^a^ ^,^ ^b^ ^,^ ^d^	0.828 (0.762–0.895)	
	Pooled mean	0.637 (0.691–0.848)	0.769 (0.691–0.848)		
PFJ stress impulse per km (MPa × s/km)	0‐kg	370.9 (325.6–416.1)	446.7 (401.5–492.0)^d^	408.8 (376.8–440.8)	Sex × load *p* < 0.001
	20.4‐kg	448.8 (395.3–502.2)^a^	561.1 (507.7–614.6)^a^ ^,^ ^d^	504.9 (467.1–542.8)	
	34.0‐kg	502.5 (438.6–566.4)^a^ ^,^ ^c^	686.8 (622.9–750.7)^a^ ^,^ ^b^ ^,^ ^e^	594.7 (549.4–639.9)	
	Pooled mean	440.7 (389.3–492.1)	564.9 (513.5–616.2)		

*Note:* Mean (95% confidence intervals). In the instance of a significant interaction, *p*‐values for the main effects and pooled means are not displayed. Please note these are unadjusted means and standard deviations.

^a^ Different from 0‐kg (*p* < 0.001).

^b^Different from 20.4‐kg (*p* < 0.001).

^c^ Different from 20.4‐kg (*p* < 0.05).

^d^ Different between sexes at same load carriage level (*p* < 0.05).

^e^ Different between sexes at same load carriage level (*p* < 0.001).
